# Epidemiological investigation of a typhoid fever outbreak in Sekhukhune District, Limpopo province, South Africa – 2017

**DOI:** 10.4102/sajid.v35i1.107

**Published:** 2020-11-16

**Authors:** Unarine B. Makungo, Tshilidzi E. Ramutshila, Mantwa C. Mabotja, Juno Thomas, Ruth Lekalakala-Mokaba, Anthony M. Smith, Joy Ebonwu, Shannon L. Williams, Jimmy Khoza, Queen Ranoto, Ntshengedzeni Muvhango, Mmatjatji Mosoma, Elizabeth Phokane, Genevie Ntshoe, Katherine Calver, Vivien Essel, Marlene F. Ngobeni, Kerrigan McCarthy

**Affiliations:** 1 Division of Public Health Surveillance and Response, National Institute for Communicable Diseases, Johannesburg, South Africa; 2 Public Health Directorate, Limpopo Department of Health, Polokwane, South Africa; 3 South African Field Epidemiology Training Program, National Institute for Communicable Diseases, Johannesburg, South Africa; 4 School of Health Systems and Public Health, University of Pretoria, South Africa; 5 School of Public Health, University of Witwatersrand, Johannesburg, South Africa; 6 Centre for Enteric Diseases, National Institute for Communicable Diseases, Johannesburg, South Africa; 7 Department of Medical Microbiology, National Health Laboratory Services, Polokwane, Limpopo, South Africa; 8 Department of Medical Microbiology, University of Limpopo, Polokwane, South Africa; 9 Department of Public Health Medicine, Limpopo Department of Health, Polokwane, South Africa; 10 Sekhukhune District, Limpopo Department of Health, Polokwane, South Africa

**Keywords:** typhoid fever, open water sources, outbreak, Limpopo, South Africa

## Abstract

**Background:**

Typhoid fever remains a public health concern in South Africa, where the risk of transmission is high because of poor access to safe water and sanitation. This study describes the investigation of typhoid fever outbreak in Limpopo province.

**Methodology:**

Following notification of laboratory-confirmed cases, a descriptive study was conducted at Sekhukhune District, Limpopo province. A suspected case was defined as any person residing in Makhuduthamaga Municipality from November 2017 to January 2018, presenting with fever and gastrointestinal symptoms. Data were collected using case investigation forms. Whole-genome sequencing (WGS) was carried out on *Salmonella* Typhi isolates and polymerase chain reaction (PCR) test was done for *Salmonella* species from water samples. Location of cases and water sources were mapped using ArcGIS mapping tool.

**Results:**

Amongst 122 cases, 54% (*n* = 66) were female and 6% (*n* = 7) laboratory-confirmed. The median age of the cases was 11 years (range 2–83 years), with 79% (*n* = 102) being children under the age of 14 years. *Salmonella* species were detected in 37% (10/27) of water samples and geographic information system (GIS) mapping showed clustering of cases in Tswaing-Kgwaripe and Vlakplaas villages. Six isolates were available for WGS analysis, with resulting data showing that five of the six isolates were genetically related. Phylogenetic analysis showed that the five isolates clustered together were genetically related showing < 22 single nucleotide polymorphisms when compared to each other.

**Conclusion:**

Molecular epidemiology of isolates suggests a common source outbreak, supported by the detection of *Salmonella* species from water sources. Consumption of water from contaminated open water sources, because of ongoing interruption of municipal water supply, was the likely cause of the outbreak. The investigation highlights the importance of consistent safe water supply and the ability of district surveillance systems to identify and contain outbreaks.

## Introduction

Water scarcity is ranked as the foremost crisis locally and globally.^1^ South Africa has made a certain degree of improvement on the establishment of sanitation and water quality infrastructures, which has played a fundamental role in the significant reduction of pathogens responsible for water and foodborne illnesses. Despite these improvements, most rural and peri-urban areas experience recurrent water and foodborne disease outbreaks, which have a negative impact on human health.^2,3^ The ongoing state of climate change accompanied by drought has become a fuel for majority of typhoid fever outbreaks in areas with poor sanitation and lack of safe water.^4,5,6^

Typhoid fever is an epidemic-prone disease caused by a gram-negative bacterium, *Salmonella* Typhi and *Salmonella* Paratyphi. It is transmitted through consumption of food or water contaminated by faeces or urine of infected persons. Typhoid fever often presents with gastrointestinal symptoms (diarrhoea, abdominal cramps) and life-threatening, enteric febrile systemic illnesses necessitating prompt antibiotic treatment to prevent complications.^7^

Typhoid fever is a life-threatening disease. Accurate diagnosis in the early stage and effective surveillance are important to ensure early detection and appropriate treatment to mitigate the spread of disease.^7^ A definitive diagnosis of typhoid fever requires the isolation of *Salmonella* Typhi from blood, bone marrow or a specific anatomical lesion. Blood culture is a recommended diagnostic test of choice given the limitations of testing other specimen types.^8^ Low- and middle-income countries with high typhoid fever endemicity are still using Widal test because it is relatively cheaper and easy to perform. However, the Widal test is not a recommended confirmatory test because of its low sensitivity, specificity and positive predictive value.^9,10^

The World Health Organization (WHO) estimates that approximately 21 million cases and 222 000 typhoid-related deaths occur annually worldwide.^11^ South Africa has observed mixed patterns of endemic disease and sporadic cases in more industrialised areas of the country through active laboratory-based surveillance, conducted by GERMS-SA of the National Institute for Communicable Diseases (NICD).^12^ The two largest typhoid fever outbreaks in the country occurred in Delmas, Mpumalanga province in 1993 and 2005, where over 1000 and 600 cases were reported, respectively.^13^ However, the number of cases and deaths has declined over the last 22 years, from 6000 cases in 1985 to 200 cases in 2002.^12,14^

On 15 November 2017, Sekhukhune District Department of Health was notified of a laboratory-confirmed *Salmonella* Typhi case from a tertiary hospital in the province. There were reports of over 20 suspected cases in one of the district hospitals in Sekhukhune District. This led to the activation of the district outbreak response team from 21 November to 31 December 2017. A descriptive epidemiological investigation was conducted to verify the existence of an outbreak, identify the source infection and implement recommendations for the control and prevention of typhoid fever spread in Sekhukhune District.

## Methods

### Study design and population

**FIGURE 1:**
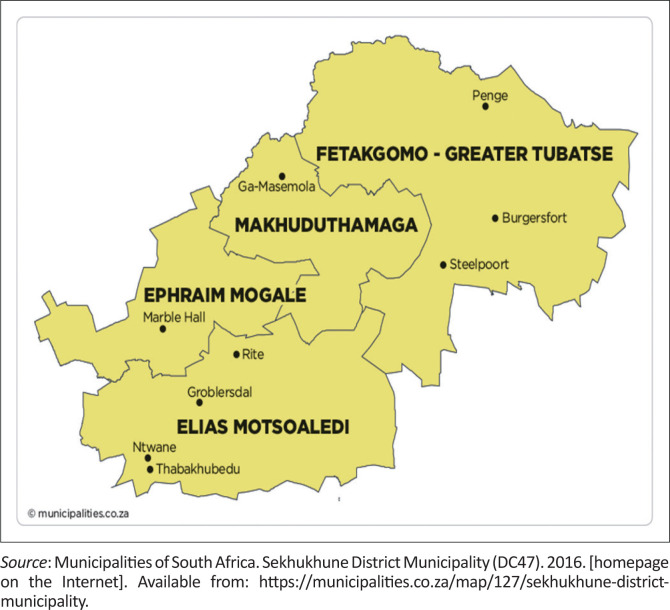
Sekhukhune map.^15^

A cross-sectional descriptive study was conducted to investigate a typhoid fever outbreak amongst 122 patients that presented with clinically compatible illness for typhoid fever at local health facilities in Makhuduthamaga Local Municipality at Sekhukhune District, Limpopo province, from mid-November 2017. Sekhukhune District is divided into five subdistricts (i.e. Greater Tubatse, Makhuduthamaga, Ephraim Mogale, Elias Motsoaledi and Fetakgomo). The geographical land distribution is 2096.60 km^2^, with a population of 274 358 according to the 2011 local municipality census (Figure 1).^15^

The following case definitions were used to identify cases. A suspected case was defined as any person who resided in Makhuduthamaga Local Municipality and presented with measured fever of +38°C with acute onset of fever and gastrointestinal symptoms during November and December 2017. A confirmed case was defined as a suspected case who had isolation of typhoidal *Salmonella* serovars from stool or blood specimens.^16^

### Data collection and tools

#### Epidemiological investigations

An epidemiological investigation was conducted by reviewing clinical records and through a standardised case investigation form (CIF). Collected data included demographics, clinical presentation, laboratory results, travel history, food preparation practices, consumption of restaurant foods, sanitation practices and source of drinking water. Data on the location of the case-patients and the sources of water were collected in January 2018 using the GIS map, focusing on the homes of case-patients, surrounding schools, crèches and sources of water.

#### Clinical and laboratory investigations

The clinicians working at the health facilities, where suspected cases presented, collected clinical samples to rule out typhoid fever. National Health Laboratory Services (NHLS) received 106 clinical specimens from 15 November to 31 December 2017. The NHLS microbiology laboratory at Pietersburg Provincial Hospital received blood (*n* = 65), stool (*n* = 6) and rectal swabs (*n* = 3) for culture. In addition, blood samples were collected and Widal tests (*n* = 32) were requested in instances where blood culture bottles were unavailable.

Six laboratory-confirmed isolates were submitted to the NICD’s Centre for Enteric Diseases (CED) for confirmation of a diagnosis of *Salmonella* Typhi and whole-genome sequencing (WGS) analysis. The CED received bacteria on Dorset-egg transport media [Diagnostic Media Products (DMP), NHLS, Johannesburg, South Africa], and then subcultured onto 5% blood agar (DMP), to check for viability and purity. Cultures were identified using standard phenotypic microbiological identification and serotyping techniques, which are briefly described here. As required, bacterial colonies were identified using the VITEK-2 COMPACT 15 automated microbial identification system (bioMérieux, Marcy-l’Étoile, France). Serotyping was performed according to the White–Kauffmann–Le Minor scheme. Antimicrobial susceptibility testing was performed using the VITEK-2 COMPACT 15 system (bioMérieux) and the Etest method (bioMérieux).

For WGS analysis of bacteria, the methodology is briefly outlined here. Genomic DNA was isolated from bacteria using the Qiagen QIAamp DNA Mini Kit (Qiagen, Hilden, Germany). DNA libraries were prepared using a Nextera XT DNA Library Preparation Kit (Illumina, San Diego, CA, USA), followed by a 2 × 300 paired-end sequencing runs with 100× coverage using Illumina MiSeq equipment. Raw data generated on the MiSeq were further analysed using tools available in the CLC Genomics Workbench Software, version 8.5 (Qiagen). Using the ‘Trim Sequences Tool’, sequence reads were trimmed to include quality trimming, ambiguity trimming and length trimming to discard reads below a length of 50 bases. Trimmed reads were assembled using the ‘*De novo* Assembly Tool’; the assembly algorithm works by using de Bruijn graphs to produce contiguous (contig) sequences (minimum contig length was set at 200 bases). For phylogenetic analysis of bacteria, assembled genome data were analysed using the ‘CSI Phylogeny 1.4’ on-line analysis pipeline available at the Center for Genomic Epidemiology (CGE) of the Technical University of Denmark (http://www.genomicepidemiology.org/). The CSI Phylogeny pipeline uses various publicly available programs and the analysis steps are briefly described as follows: assembled genome data is aligned against a reference genome and single nucleotide polymorphisms (SNPs) are called; SNPs are filtered and qualified; final qualified SNPs for each genome are concatenated to an alignment; and phylogeny is then inferred based on a comparison of SNP alignments of strains. Single nucleotide polymorphisms were called by alignment and referencing against a South African strain isolated in 2016 (reference number TCD981492). Single nucleotide polymorphism alignments were analysed with iTOL software to generate phylogenetic maximum-likelihood trees.

#### Environmental investigations

Water sampling sites were guided by information from case-patients’ interviews. Water samples were collected by environmental health practitioners (EHPs) and tested for *Salmonella* species using polymerase chain reaction (PCR) method at the Council for Scientific and Industrial Research (CSIR) laboratory.

#### Geographic information system mapping

Geographic information system (GIS) mapping of the case-patients’ locations and water sources was conducted by a trained NICD team and Sekhukhune Department of Health district staff (health promotion coordinator and ward-based outreach team), which was led by a GIS specialist, at all affected villages. Demographic and Global Positioning System (GPS) coordinates were collected and color-coded points were used to indicate cases (confirmed, suspected) and water sources tested for *Salmonella* species.

#### Health promotion activities

Health Promotion activities were conducted during the outbreak through door-to-door consultations, community gatherings at the 13 affected villages, local radio station and primary health facilities. Ward committee councillors were engaged by Sekhukhune outbreak response team to facilitate awareness on food and waterborne illness.

#### Clinical in-service training activities

Typhoid fever clinical management training was conducted by District Health Special Program Unit and NICD staff during and after the outbreak. The training was conducted because of observed non-uniformity and challenges on diagnosis and treatment of typhoid fever cases by clinicians.

### Data analysis

**FIGURE 2:**
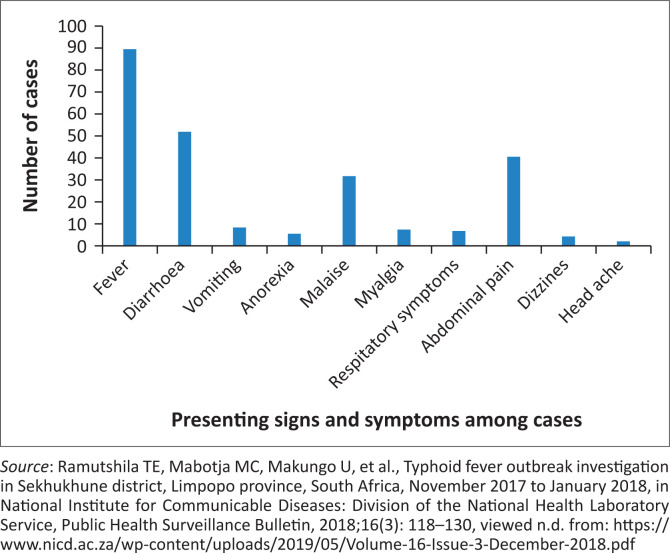
Presenting signs and symptoms amongst all cases, Sekhukhune District, Limpopo province, November – December 2017.

Categorical data were summarised as proportions. Descriptive statistics (median and range) were used to describe continuous variables. The epidemic curve was created using the date of onset of symptoms (Figure 2).^31^ Data management and analysis were performed using Microsoft Excel 2016.

### Ethical consideration

The study was approved by the Limpopo Provincial Research Ethics Committee Organization: Office of the Premier (Clearance number: LPREC/19/2018: PG).

## Results

### Epidemiological findings

**FIGURE 3:**
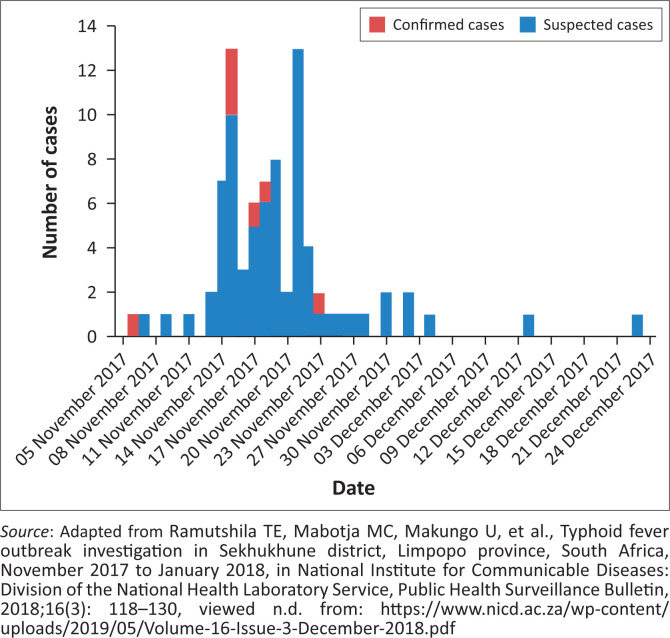
Epidemic curve of typhoid fever cases (suspected and confirmed) by date of onset, Sekhukhune District, Limpopo province, November – December 2017 (*N* = 122).

Between 15 November and 31 December 2017, a total of 122 cases were reported, of which 6% (7/122) were laboratory-confirmed for *Salmonella* Typhi by blood culture. The patient characteristics are described in Table 1. The median age of the cases was 11 years (range 2–83 years), with most cases between 5 and 14 years (64%, 78/122). Females accounted for 54% (66/122) of the cases. Majority of cases were from Tswaing village (67%, 82/122), with four laboratory-confirmed cases. Most case-patients (67%, 82/122) reported obtaining water for domestic use from irrigation furrows and other untreated water sources because of interrupted municipal water supply (Table 1).^31^ The common presenting symptoms were fever (47%, 90/122), diarrhoea (43%, 52/122) and abdominal pain (34%, 41/122). The epidemic curve of all the cases by date of onset is presented in Figure 3.^31^ The epidemic curve suggests the outbreak started in early November and peaked between the second and third weeks.

**TABLE 1:** Characteristics of typhoid fever cases, Sekhukhune District, Limpopo province, November – December 2017 (*N* = 122).

Characteristic	All cases (*n* = 122)		Suspected cases (*n* = 115)		Confirmed cases (*n* = 7)	
	*n*	%	*n*	%	*n*	%
**Age group(years)**						
≤ 4	14	11	13	11	1	14
5–14	78	64	76	66	2	29
15–49	25	21	22	19	3	43
≥ 50	5	4	4	4	1	14
**Gender**						
Female	66	54	62	54	5	71
Male	56	46	53	46	2	29
**Residental area**						
Apelcross	2	2	1	1	1	14
Ga-masemola	6	5	6	5	0	0
Gaphaahla	1	1	0	0	1	14
Tswaing	82	67	78	68	4	58
Vlakplaas	12	10	12	10	0	0
Strydkraal	11	9	10	9	1	14
Other	8	6	8	7	0	0
**Health facility consulted**						
A	94	77	92	80	2	29
B	27	22	22	19	5	71
C	1	1	1	1	0	0
**Water Sources**						
Stream	9	77	7	6	2	29
Irrigation furrow	70	57	67	58	3	43
Water tank	1	1	1	1	0	0
Borehole	2	2	2	2	0	0
Communal taps	2	2	1	1	1	14
Well	1	1	0	0	1	14
Unknown	37	30	37	32	0	0
**Type of toilet**						
Pit latrine	110	90	103	90	7	100
Unknown	22	10	22	10	0	0

*Source:* Ramutshila TE, Mabotja MC, Makungo U, et al., Typhoid fever outbreak investigation in Sekhukhune district, Limpopo province, South Africa, November 2017 to January 2018, in National Institute for Communicable Diseases: Division of the National Health Laboratory Service, Public Health Surveillance Bulletin, 2018;16(3): 118–130, viewed n.d. from: https://www.nicd.ac.za/wp-content/uploads/2019/05/Volume-16-Issue-3-December-2018.pdf

### Laboratory analysis of isolates

**FIGURE 4:**
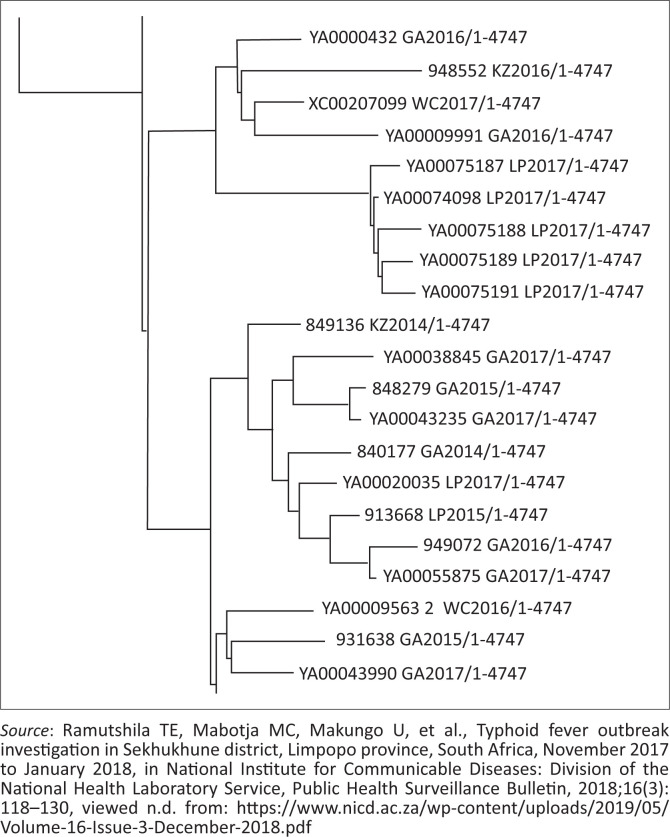
Snapshot from a maximum likelihood phylogenetic tree drawn using single nucleotide polymorphism alignments from whole-genome sequencing data of *Salmonella* Typhi isolates from South Africa. The blocked region highlights a cluster of related isolates sourced at the Sekhukhune District, Limpopo province, November to January 2018.

Six isolates were available for WGS analysis, with resulting data showing that five of the six isolates were genetically related. Phylogenetic analysis showed that the five related isolates clustered together, with isolates showing <22 SNPs when compared to each other. Of the six isolates sent for WGS, five isolates that were obtained from cases in Tswaing and Strydkraal villages were genetically related (<22 SNPs), as shown in Figure 4.^31^ A single, un-clustered strain was closely related to the 2016 Zimbabwe typhoid fever outbreak. All six isolates were susceptible to ciprofloxacin and ceftriaxone.

### Environmental findings

**FIGURE 5:**
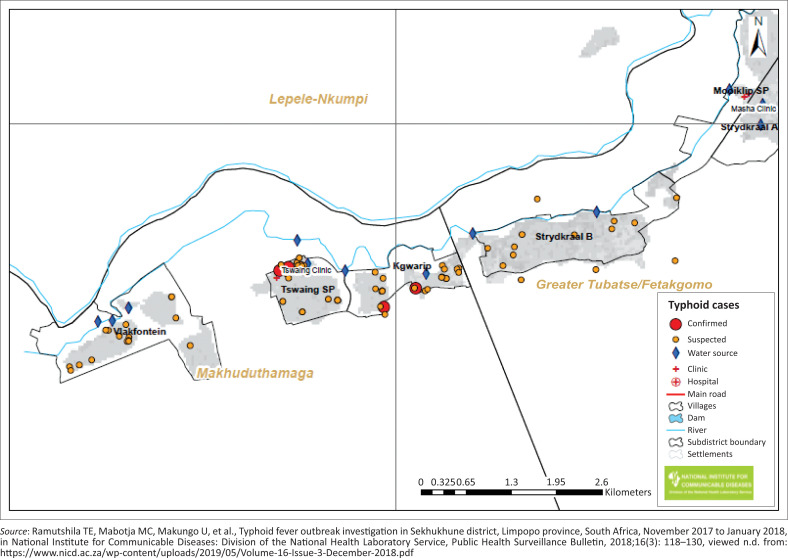
Geographic distribution of suspected and confirmed typhoid fever cases and water sources, Sekhukhune District, Limpopo province, November – December 2017.

Of the seven confirmed cases, 86% (*n* = 6) reported using water from the open water sources. Pathogenic *Salmonella* species were detected in 10/27 (37%) samples from water sources in the district. The GIS mapping showed clustering of cases in Village S, with 4/7 (57%) laboratory-confirmed cases and 78/122 (64%) suspected cases (Figure 5).

## Discussion

This outbreak investigation discovered that rural communities are still at a disadvantage for basic human rights such as safe water and sanitation despite national infrastructure plans in place. Consumption of contaminated water from untreated open sources was the likely driver of infection in this outbreak. This was evidenced by six of the seven laboratory-confirmed cases where water was reported to have been used from open sources for drinking and other household activities. A relation between typhoid fever and contaminated water sources has been identified in other studies.^17,18^ Unsafe disposal of excreta and solid waste might have led to the contamination of open water sources, which has been reported in other settings.^19,20^ The geographical location of water sources sampled in our investigation is in close proximity to villages where cases are clustered. The use of open source drinking water by residents of affected villages is the most likely factor to have resulted in the typhoid fever outbreak. These findings are consistent with results of previous similar outbreak investigations.^13,19,21^

The sudden increase in cases after the start of the investigation was likely because of active community outreach and awareness about typhoid fever signs, symptoms and prevention. A steady decline in cases from the last week of November was probably because of heightened community awareness, high suspicion index at treating hospitals and fixing of the broken down water plant by the local municipality, as well as provision of free water purification products and clean water. This is indicative of how a multisectoral approach impacts mitigation of disease outbreaks.^5,22^ The investigation highlights the importance of consistent safe water supply, and the ability of district surveillance systems to identify and contain outbreaks.

A high burden of typhoid fever is often observed mostly in the paediatric population in low- and middle-income countries.^23,24^ In our study, 75% (92/122) of the cases were under 15 years of age. Although no deaths were reported during this outbreak, a high burden of typhoid fever in children is a public health concern, as there is a > 4 time’s risk of mortality because of increased risk of complications and prolonged hospitalisation that has been observed in other settings.^25,26^ The high burden of typhoid fever amongst children in this study specifies the high-risk population but also enables policymakers and municipal service delivery sectors to make informed decisions on prioritisation of safe water supply in schools and the community in general.

Knowledge about typhoid fever clinical cases management and public health response is fundamental to how well typhoid outbreaks are contained and prevented. Definitive diagnosis of typhoid fever, such as isolation of *Salmonella* Typhi from blood culture, is largely available and affordable in South Africa.^16^ The use of Widal test may result in misdiagnosis and mismanagement of patients.^9,10,27^ During the investigation period, in-service training on clinical management and public health response of typhoid fever were conducted in the district after it became known that there was no uniformity in patient management amongst clinicians. Typhoid fever management guidelines were distributed to all hospitals to ensure the proper management of patients.^16,28^

## Recommendations

The following recommendations were given to the provincial and local authorities.

- Department of Health, Department of Water Affairs and municipality should form an intersectoral committee that facilitates communication between departments and ensures that challenges pertaining to access to clean water and sanitation are addressed promptly.
- Purification and chlorination of public water supplies should be monitored by the intersectoral health, water and sanitation committee.
- There should be continuous community health education and awareness on water, sanitation and hygiene practices.
- Case management should include contact tracing with an investigation for asymptomatic carriage, documentation of eradication of carriage, and restrictions on food handling practices amongst laboratory-confirmed cases until at least three consecutive negative stool cultures have been taken.
- Health care workers should complete standardised CIFs to ensure that high quality data is collected.
- Department of Health should develop a case control outbreak investigation protocol to guide field workers and to ensure more robust future investigations.

## Limitations

The study had a few limitations. The PCR-based test used by CSIR to test environmental water samples detects all *Salmonella enterica* subspecies as well as *Salmonella bongori,* but it cannot distinguish between typhoidal and non-typhoidal *Salmonella* species.^29,30^

Because of the nature of the cross-sectional study design, we could not determine risk factors that contributed to typhoid fever infection.

## Conclusion

This descriptive study highlights the importance of outbreak investigation and management of typhoid fever cases to ensure disease control. The observed decline of cases further highlights that access to clean water, a multisectoral approach and communication amongst different stakeholders in conjunction with training of health professionals on diagnosis and management of waterborne diseases have an impact on the management of epidemic-prone diseases.

## References

[1] Cole MJ, Bailey RM, Cullis JD, New MG. Spatial inequality in water access and water use in south Africa. Water Policy. 2018;20(1):37–52. 10.2166/wp.2017.111

[2] Levy K, Smith SM, Carlton EJ. Climate change impacts on waterborne diseases: Moving toward designing interventions. Curr Environ Health Rep. 2018;5(2):272–282. 10.1007/s40572-018-0199-7 29721700 PMC6119235

[3] Zolnikov TR. Climate change: Water and sanitation. In: Brears RC, editor. Climate resilient water resources management [homepage on the Internet]. Cham: Springer International Publishing; 2018 [cited 2020 Aug 29]. p. 5–14 (Palgrave Studies in Climate Resilient Societies). Available from: 10.1007/978-3-319-78896-8_2

[4] Meissner R, Steyn M, Moyo E, et al. South African local government perceptions of the state of water security. Environ Sci Policy. 2018;87(1):112–127. 10.1016/j.envsci.2018.05.020

[5] Bridge JW, Oliver DM, Chadwick D, et al. Engaging with the water sector for public health benefits: Waterborne pathogens and diseases in developed countries. Bull World Health Organ. 2010;88(1):873–875. 10.2471/BLT.09.072512 21076571 PMC2971506

[6] Mogasale VV, Ramani E, Mogasale V, Park JY, Wierzba TF. Estimating typhoid fever risk associated with lack of access to safe water: A systematic literature review. J Environ Public Health. 2018. Article ID: 9589208. 10.1155/2018/9589208 PMC6076975 30174699

[7] Stadtländer CK. Control of communicable diseases manual. 18th ed. In: Heymann DL, editor. Washington DC: American Public Health Association; 2004.

[8] Crump JA, Sjölund-Karlsson M, Gordon MA, Parry CM. Epidemiology, clinical presentation, laboratory diagnosis, antimicrobial resistance, and antimicrobial management of invasive Salmonella infections. Clin Microbiol Rev. 2015;28(4):901–937. 10.1128/CMR.00002-15 26180063 PMC4503790

[9] Ley B, Mtove G, Thriemer K, et al. Evaluation of the Widal tube agglutination test for the diagnosis of typhoid fever among children admitted to a rural hdospital in Tanzania and a comparison with previous studies. BMC Infect Dis. 2010;10(1):180. 10.1186/1471-2334-10-180 20565990 PMC2898821

[10] Mawazo A, Bwire GM, Matee MIN. Performance of Widal test and stool culture in the diagnosis of typhoid fever among suspected patients in Dar es Salaam, Tanzania. BMC Res Notes. 2019;12(1):316. 10.1186/s13104-019-4340-y 31167646 PMC6551910

[11] World Health Organization. Typhoid [homepage on the Internet]. 2018 [cited 2020 Aug 28]. Available from: https://www.who.int/immunization/diseases/typhoid/en/

[12] Keddy KH, Smith AM, Sooka A, et al. The burden of typhoid fever in South Africa: The potential impact of selected interventions. Am J Trop Med Hyg. 2018;99(3_Suppl):55–63. 10.4269/ajtmh.18-0182 30047360 PMC6128358

[13] Waner S, Kfir R, Idema GK, et al. Waterborne outbreak of typhoid fever in Delmas. S Afr J Epidemiol Inf. 1998;13(1):53–57.

[14] National Department of Health. Typhoid cases in South Africa and Gauteng Province [homepage on the Internet]. 2016 [cited 2020 Aug 30]. Available from: https://www.google.com/url?sa=t&rct=j&q=&esrc=s&source=web&cd=&ved=2ahUKEwj_yva3m6fsAhV5UhUIHSK-B8IQFjAAegQIARAC&url=https%3A%2F%2Fpmg.org.za%2Ffiles%2F160309Typhoid.pptx&usg=AOvVaw2dseSTB2kQw6KSH5Rdejal

[15] Municipalities of South Africa. Sekhukhune District Municipality (DC47). 2016 [homepage on the Internet]. Available from: https://municipalities.co.za/map/127/sekhukhune-district-municipality

[16] Typhoid recommendation for diagnosis, management, and public health response [homepage on the Internet]. 2016 [cited 2020 Aug 29]. Available from: http://www.nicd.ac.za/assets/files/guideline

[17] Swaddiwudhipong W. A common-source water-borne outbreak of multi-drug-resistant typhoid fever in a rural Thai community. J Med Assoc Thai. 2001;84(11):1513–1517. 11853291

[18] Bhunia R, Hutin Y, Ramakrishnan R, Pal N, Sen T, Murhekar M. A typhoid fever outbreak in a slum of South Dumdum municipality, West Bengal, India, 2007: Evidence for foodborne and waterborne transmission. BMC Public Health. 2009;9(1):115. 10.1186/1471-2458-9-115 19397806 PMC2683821

[19] Davis WW, Chonzi P, Masunda KP, et al. Notes from the field: Typhoid fever outbreak—Harare, Zimbabwe, October 2016–March 2017. MMWR Morb Mortal Wkly Rep. 2018;67(11):342. 10.15585/mmwr.mm6711a7 29565843 PMC5868204

[20] A large and persistent outbreak of typhoid fever caused by consuming contaminated water and street-vended beverages: Kampala, Uganda. BMC Public Health [homepage on the Internet]. 2015 [cited 2020 Aug 29]. Available from: https://bmcpublichealth.biomedcentral.com/articles/10.1186/s12889-016-4002-0 10.1186/s12889-016-4002-0 PMC5216563 28056940

[21] Farooqui A, Khan A, Kazmi SU. Investigation of a community outbreak of typhoid fever associated with drinking water. BMC Public Health. 2009;9(1):476. 10.1186/1471-2458-9-476 20021691 PMC2804617

[22] Raviglione M, Maher D. Ending infectious diseases in the era of the Sustainable Development Goals. Porto Biomed J. 2017;2(5):140–142. 10.1016/j.pbj.2017.08.001 32258607 PMC6806806

[23] Roy JS, Saikia L, Medhi M, Tassa D. Epidemiological investigation of an outbreak of typhoid fever in Jorhat town of Assam, India. Indian J Med Res. 2016;144(4):592–596. 10.4103/0971-5916.200902 28256469 PMC5345307

[24] Antillón M, Warren JL, Crawford FW, et al. The burden of typhoid fever in low-and middle-income countries: A meta-regression approach. PLoS Negl Trop Dis. 2017;11(2):e0005376. 10.1371/journal.pntd.0005376 28241011 PMC5344533

[25] Azmatullah A, Qamar FN, Thaver D, Zaidi AK, Bhutta ZA. Systematic review of the global epidemiology, clinical and laboratory profile of enteric fever. J Glob Health. 2015;5(2):020407. 10.7189/jogh.05.020407 26649174 PMC4672836

[26] Singh KG, Sundar JS. A study on clinical profile of typhoid fever at Government General Hospital, Nizamabad, Telangana, India. Int J Contemp Pediatr. 2019;6(6):2642. 10.18203/2349-3291.ijcp20194746

[27] Wam EC, Arrey CN, Sama LF, Agyingi LA, Wam AN. Comparative study on the use of Widal test to stool culture in the laboratory diagnosis of typhoid fever in Holy Family Hospital Akum, North West Region of Cameroon. Open Microbiol J. 2019;13(1):73–80. 10.2174/1874285801913010073

[28] THD. Environmental scarcity and violent conflict: The case of South Africa [homepage on the Internet]. Thomas Homer-Dixon; 1998 [cited 2020 Aug 29]. Available from: https://homerdixon.com/environmental-scarcity-and-violent-conflict-the-case-of-south-africa/

[29] Malorny B, Hoorfar J, Bunge C, Helmuth R. Multicenter validation of the analytical accuracy of Salmonella PCR: Towards an international standard. Appl Environ Microbiol. 2003;69(1):290–296. 10.1128/aem.69.1.290-296.2003 12514007 PMC152403

[30] Gal-Mor O, Boyle EC, Grassl GA. Same species, different diseases: How and why typhoidal and non-typhoidal Salmonella enterica serovars differ. Front Microbiol. 2014;5(1):391. 10.3389/fmicb.2014.00391 25136336 PMC4120697

[31] Ramutshila TE, Mabotja MC, Makungo U, et al., Typhoid fever outbreak investigation in Sekhukhune district, Limpopo province, South Africa, November 2017 to January 2018, in National Institute for Communicable Diseases: Division of the National Health Laboratory Service, Public Health Surveillance Bulletin, 2018;16(3): 118–130, viewed n.d. from: https://www.nicd.ac.za/wp-content/uploads/2019/05/Volume-16-Issue-3-December-2018.pdf

